# ABT-869, a promising multi-targeted tyrosine kinase inhibitor: from bench to bedside

**DOI:** 10.1186/1756-8722-2-33

**Published:** 2009-07-30

**Authors:** Jianbiao Zhou, Boon-Cher Goh, Daniel H Albert, Chien-Shing Chen

**Affiliations:** 1 Department of Medicine, Yong Loo Lin School of Medicine, National University of Singapore, Singapore; 2 Cancer Science Institute of Singapore, National University of Singapore, Singapore; 3 Department of Hematology and Oncology, National University Hospital, Singapore; 4 Cancer Research, Abbott Laboratories, Abbott Park, Illinois, USA; 5 School of Medicine, Division of Hematology and Oncology, Loma Linda University, Loma Linda, California, USA

## Abstract

Tyrosine Kinase Inhibitors (TKI) have significantly changed the landscape of current cancer therapy. Understanding of mechanisms of aberrant TK signaling and strategies to inhibit TKs in cancer, further promote the development of novel agents.

ABT-869, a novel ATP-competitive receptor tyrosine kinase inhibitor is a potent inhibitor of members of the vascular endothelial growth factor (VEGF) and platelet derived growth factor (PDGF) receptor families. ABT-869 showed potent antiproliferative and apoptotic properties *in vitro *and in animal cancer xenograft models using tumor cell lines that were "addicted" to signaling of kinases targeted by ABT-869. When given together with chemotherapy or mTOR inhibitors, ABT-869 showed at least additive therapeutic effects. The phase I trial for ABT-869 was recently completed and it demonstrated respectable efficacy in solid tumors including lung and hepatocellular carcinoma with manageable side effects. Tumor cavitation and reduction of contrast enhancement after ABT-869 treatment supported the antiangiogenic activity. The correlative laboratory studies conducted with the trial also highlight potential biomarkers for future patient selection and treatment outcome.

Parallel to the clinical development, *in vitro *studies on ABT-869 resistance phenotype identified novel resistance mechanism that may be applicable to other TKIs. The future therapeutic roles of ABT-869 are currently been tested in phase II trials.

## Introduction

Receptor tyrosine kinases (RTKs) and protein phosphatases control reversible protein phosphorylation [1,2]. This process mediates critical signaling transduction between cell and extracellular stimulation, including survival, growth and differentiation. Dysregulation of RTK signaling pathways has been correlated with the progression of cancers with different histological origins [1]. For example, amplification of the HER2 gene is observed in ~30% of breast cancer biopsies and forms the basis for the use of trastuzumab (Herceptin, Genentech, Inc, California) to treat breast cancer patients.

The common molecular mechanisms underlying such aberrant activities are point mutation, duplication, and amplification of the RTK, which leads to gain-of-function and consecutive activation of the kinases in general. The fms-like tyrosine kinase 3 (FLT3) is a class III RTK family and shares strong structural similarity with other family members including receptors for platelet-derived growth factors A (PDGFRA) and B (PDGFRB), the colony-stimulating factor 1 receptor (CSF1-R) and steel factor receptor (KIT) [3-5]. FLT3 mutations are identified in about one-third of adult acute myeloid leukemia (AML) [6-10]. The interactions between the vascular endothelial growth factors (VEGF) and their receptors (VEGFRs) are crucial for angiogenesis [11,12]. The expression of VEGF and its receptors are detected in most of solid tumors and hematological malignancies [13]. Overexpression of VEGF and/or it's receptor VEGFR2 contributes to invasiveness and metastasis of breast, lung, prostate, renal-cell, colon cancers and hepatocellular carcinoma [11,12]. In AML, a number of studies have demonstrated that an autocrine/paracrine pathway between VEGF and its receptors are involved in poor survival of a subset of patients and progression of the disease [14-17]. This evidence underpins an important discovery in the molecular biology of cancer that histological different types of cancer could share the same dysregulated signaling pathway(s) and one particular type of cancer could have multiple genetic abnormalities. Therefore, there has been great interest in discovering compounds targeting multiple RTKs with the rationale of potential superior antitumor activity for a variety of cancer types.

ABT-869, a novel ATP-competitive RTK inhibitor, is active against all VEGFRs and PDGFR families, but minimally active against unrelated RTKs and cytosolic tyrosine kinases and serine/threonine kinases [18]. The goals of this article are to summarize the published data on preclinical and clinical development of ABT-869, an orally active multi-targeted RTK inhibitor in the treatment of leukemia and solid tumors. Secondly, various strategies and rationale as well as mechanistic studies of combining ABT-869 with other agents will be reviewed. Lastly, we discuss the potential drug resistance issue in ABT-869 therapy based on our laboratory's published data. ABT-869 is under active clinical development primarily in solid tumors and early phase data and ongoing phase II studies will be reviewed.

## The chemical structure and target selection of ABT-869

**Figure 1:**
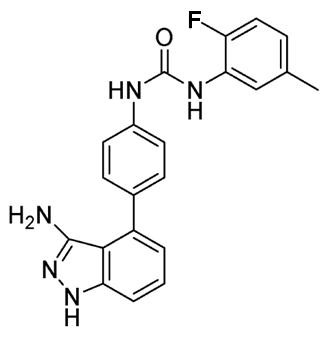
**The chemical structure of ABT-869**: N- [4-(3-amino-1H-indazol-4-yl)phenyl]- N1-(2-fluoro-5-methylphenyl) urea.

**Figure 2:**
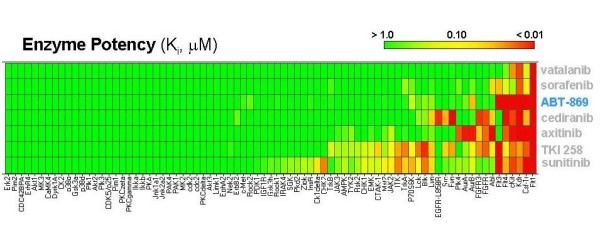
**Kinase inhibition profile of ABT-869 against a broader range of kinases**.

ABT-869 was discovered in Abbott Laboratories (Abbott Park, IL, USA) through a structure-based rational design, by incorporating an N, N'-diaryl urea moiety at the C4-position of 3-aminodazole (Figure 1) [19]. The molecular weight of ABT-869 is 375.4. ABT-869 shows potent efficacy to inhibit all the members of VEGFR and PDGFR family with nanomolar range of IC_50_, but much less activity to other nonrelated tyrosine kinase (Table 1) [18]. The selectivity profile of ABT-869 against a broader range of kinases is illustrated in Figure 2. In comparison to 5 other multitargeted RTK inhibitors (PTK787 [Vatalanib^®^, Novartis-Schering AG], AG013736 [Axitinib^®^, Pfizer], BAY43-9006 [Nexavar^®^, Bayer], CHIR258 [Chiron], and SU11248 [Sutent^®^, Pfizer]) [19], that have undergone clinical development, ABT-869 inhibited a broader number of kinases relevant to the VEGF signaling pathway. AG013736, CHIR258, and SU11248 are also active against most of the targeted kinases but these inhibitors demonstrate more off-target activity than ABT-869 [18].

**Table 1:** Kinase inhibition profile of ABT-869 (with permission adapted from Molecular Cancer Therapeutics 2006;5:995–1006)

**Related RTK^a^**		**Non-related TK^a^**		**Ser/Thr Kinases^b^**	
**Kinase**	**IC_50 _(nM)**	**Kinase**	**IC_50 _(nM)**	**Kinase**	**IC_50 _(nM)**

KDR	8	SRC	> 50,000	AKT	> 50,000
FLT1	3	IGFR	> 50,000	SGK	940
FLT4	40	INSR	> 50,000	CDC2	9,800
PDGFRα	29	LCK	38,000	PKA	5,900
PDGFRβ	25	EGFR	> 50,000		
CSF-1R	5	HCK	> 50,000		
KIT	20	CMET	> 50,000		
FLT3	10	LYN	> 20,000		
TIE2	170	FYN	> 50,000		
RET	1,900	FGR	> 50,000		
FGFR	> 12,500				

a. IC_50 _values determined at an ATP concentration of 1 mM.

b. IC_50 _values determined at an ATP concentration of 5 to 10 μM.

**Figure 3:**
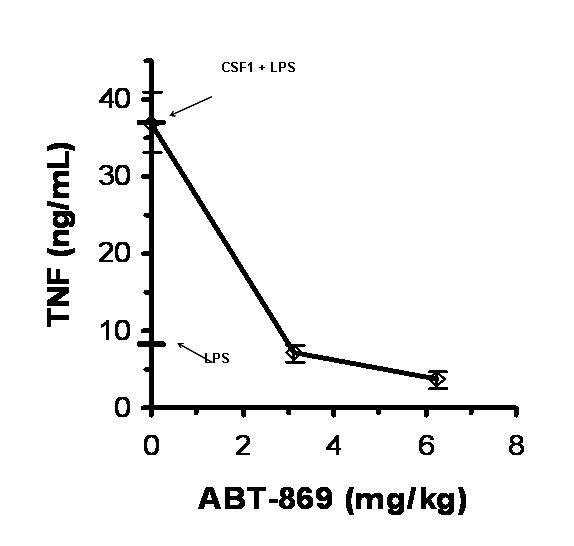
**Inhibition of CSF1-primed LPS-induced TNF release**. Mice were given ABT-869 (PO) at the indicated dose and 45 minutes later primed with CSF1 (1.8 μg IP). After 3.25 hours, LPS (300 μg IP) was administered. Serum TNF, expressed as mean ± SEM (n = 6), was assessed 1.5 hours later. CSF1 increased serum TNF induced by LPS by >4 fold (8 vs 37 ng/mL).

Another potentially important aspect of the distinctive activity profile of ABT-869 is the molecule's activity against CSF1R [20]. This activity is manifested as potent inhibition of CSF-1R signaling in macrophage-derived cells [21]. In vivo activity of ABT-869 for inhibiting CSF1R-mediated responses is exemplified by results illustrated in Figure 3 showing the effect of oral administration of ABT-869 on CSF1 priming of LPS-induced TNF release in mice. This activity may contribute to the anti-tumor activity of ABT-869 in cancer models where elevated levels of inflammatory tumor-associated macrophages drive tumor progression.

## Nonclinical in vivo activity of ABT-869

**Figure 4:**
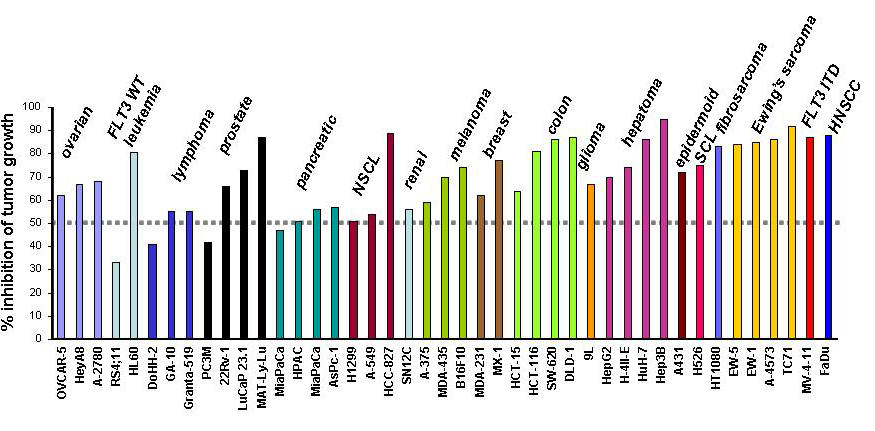
**Efficacy of ABT-869 in representative xenografts**. Efficacy was defined as percent of tumor size relative to vehicle-treated remaining after 3–4 weeks of dosing ABT-869 (10–25 mg/kg/day).

Initial nonclinical studies demonstrated potent antiproliferative and apoptotic effects of ABT-869 on cancer cells whose proliferation is dependent on mutant kinases, such as FLT3 [18,20,22]. ABT-869 given orally was effective in multiple in vivo human xenograft tumor growth models and showed *in vivo *mechanism-based targeting, including acute myeloid leukemia with FLT3 mutation (MV4–11), highly angiogenic fibrosarcoma (HT1080), small cell lung carcinoma (H526, known to express KIT), colon adenocarcinoma (DLD-1), epidermoid carcinoma (A431) and breast cancinoma (MX-1). In addition to flank xenografts, ABT-869 has demonstrated dose dependant efficacy in orthotopic tumor growth models with the breast carcinoma cell lines MDA-231 (epithelial) and MDA-435LM (ductal) as well as a rat glioma cell line (9L). ABT-869 was also efficacious at inhibiting the growth of prostate cancer cells in a bone environment, thereby demonstrating potential therapeutic utility in a metastases setting [23]. A summary of activity in these and other tumor models is presented in Figure 4.

**Figure 5:**
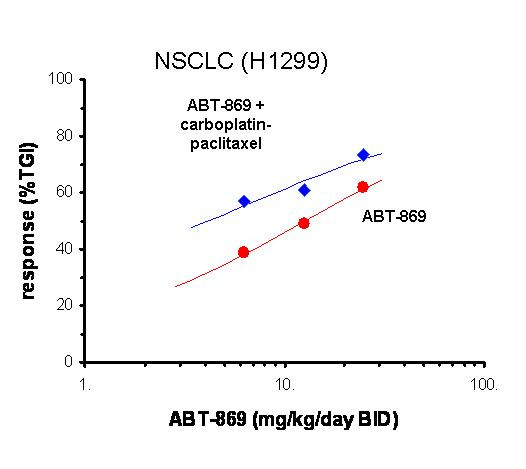
**Efficacy of ABT-869 in combination with carboplatin-paclitaxel in a NSCLC xenograft**. ABT-869 was administered orally at the indicated dose for 3 weeks and carboplatin-paclitaxel was administered weekly (IP and IV respectively) beginning 3 weeks after inoculation of H1299 cells into the flank of SCID/beige mice. Percent inhibition of tumor size relative to vehicle treated control was calculated at the end of the study is indicated in parentheses in the legend.

In addition to single agent activity ABT-869 also exhibited antitumor activity when given in combination with chemotherapy agents, including: carboplatin, cisplatin, docetaxel, gemcitabine, irinotecan, paclitaxel, rapamycin, TMZ and Ara-C [18,22,24,25]. The effect of combination therapy with carboplatin-paclitaxel (dosed concurrently) on the dose-dependent activity of ABT-869 in a NSCLC model response is shown in Figure 5. This response to combination therapy is typical in that it reflects an increase in efficacy with no increase in overall toxicity. However, the outcome of combination therapy can be somewhat sequence-dependent, as is discussed below.

In light of its preclinical activity profile, ABT-869 underwent the industrial standard pre-clinical toxicology, metabolism, and pharmacology studies and the compound was deemed to be suitable to further clinical development (see below).

## Nonclinical studies of ABT-869 and in combination with chemotherapy in acute myeloid leukemia with and without FLT-3 mutations

**Figure 6:**
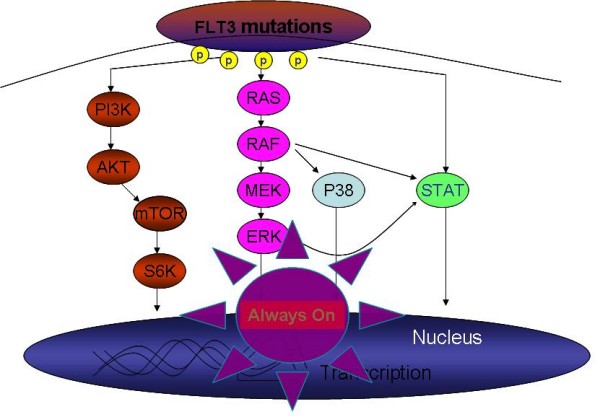
**The FLT3-ITD signaling pathways**. The presence of FLT3-ITD induces ligand-independent receptor dimerization and activates three major signaling pathways including PI3K/AKT, MAPK and STAT5 pathways. These signalings are transferred to nucleus, which lead to the transcription of genes involved in cell proliferation and survival.

Approximately, 25% of AML patients have acquired FLT3 internal tandem duplications (FLT3-ITDs), varying from 3 to ≥ 400 base pairs in the juxtamembrane domain, and 7% of AML patients harbor activating point mutations in the second kinase domain (FLT3-TK) [7-10]. FLT3 mutations therefore represent the most common genetic alteration in AML and therefore, have been targeted for therapeutic agent development. Patents with FLT3-ITD are usually associated with poor outcome, but the prognosis of FLT3-TK mutation remains inconclusive [7-10]. FLT3-ITD mutations trigger strong autophosphorylation of the FLT3 kinase domain, and constitutively activate several downstream effectors such as the PI3K/AKT pathway, RAS/MAPK pathway, and the STAT pathway, mainly STAT5 (Figure 6). Oncogenic protein kinase PIM1 also is up-regulated by FLT3-ITD. These rampant signaling pathways are wired to promote uncontrolled cell survival and proliferation, leading to transformation of leukemia [26].

For leukemia cell lines with FLT3-ITD such as MV4–11 and MOLM-14, ABT-869 potently inhibits their proliferation at IC_50 _less than 10 nM [22,27]. ABT-869 also induces dose-dependently G_1 _cell cycle arrest and apoptosis in these FLT3-ITD positive cells [22,27]. Analysis of key cell cycle regulators reveals that simultaneous terminal reduction of cyclins D and E, the key G_1_/S cyclins, and progressive increases in cyclin dependent kinase inhibitors (CDKIs) p21^waf1/Cip^, p27^kip1 ^contributed to the blockage of G_1_/S progression induced by ABT-869 [22]. ABT-869 increases the expression of a few proapoptotic proteins including BAD, BAK and BID, and decreases the pro-survival molecule Bcl-xL. Cleaved BID and PARP, a hallmark of apoptosis, is evident [22].

ABT-869, as predicted from its kinase inhibition profile, targets the FLT3 signaling pathway. In MV4–11 cells, ABT-869 inhibits phosphorylation of FLT3 receptor (p-FLT3), as well as downstream signaling effectors p-AKT, p-ERK, p-STAT5 and PIM-1 kinase at a concentration of 1 nM [22,27]. Importantly, ABT-869 has been shown to effectively inhibit colony formation of primary AML bone marrow cells at 100 nM, but no inhibition on normal human bone marrow progenitor cells up to 1 μM, suggesting ABT-869 is not toxic to normal bone marrow cells [27]. In a mice bone marrow engraftment model of MV4–11 cells, ABT-869 treatment significantly prolonged survival and reduced leukemic burden (CD45+ human cells) in a dose-dependent fashion when compared to vehicle control treatment [27].

However, considering the complexity of the disease, ABT-869 as a single agent is unlikely to deliver complete or lasting responses in AML. We demonstrated that ABT-869 also produces synergistic antileukemic effect with chemotherapy in a sequence dependent manner [22]. This sequence-specific synergism was also demonstrated with another FLT3 inhibitor, CEP-701 (Lestaurtinib^®^, Cephalon, Inc., Frazer, PA, USA) [28]. For simultaneous treatment in MV4–11 and MOLM-14 cells, combination of lower doses of ABT-869 and cytosine arabinoside (Ara-C) generates an additive or mildly synergistic interaction. All of the combinations of ABT-869 and Doxorubicin (Dox) results in synergistic effects. However, pretreatment with ABT-869 antagonizes the cytotoxicity of Ara-C and Dox [22]. In contrast, chemotherapy (either Ara-C or Dox) followed by ABT-869 produces significant synergism on inhibition of proliferation and induction of apoptosis in MV4–11 and MOLM-14 cells, as well as primary patient AML cells with FLT3-ITD mutations [22]. In a MV4–11 tumor xenograft model, combination of Ara-C at 15 mg/kg/day for 4 days and ABT-869 at 15 mg/kg/day results in faster reduction of tumor burden compared to ABT-869 treatment alone. Importantly, no adverse side effect is observed in the combination treatment group in terms of behavior or body weight changes [22]. Low density array (LDA) analysis reveals that inhibition of cell cycle related genes and MAPK pathway play an important role in the synergistic mechanism. Particularly, Cyclin D1 (CCND1) and Moloney murine sarcoma viral oncogene homolog (c-Mos) were the two most significantly downregulated genes [22]. Collectively, these studies help to define the optimal combination sequence of chemotherapy and ABT-869 for clinical trials in AML.

**Figure 7:**
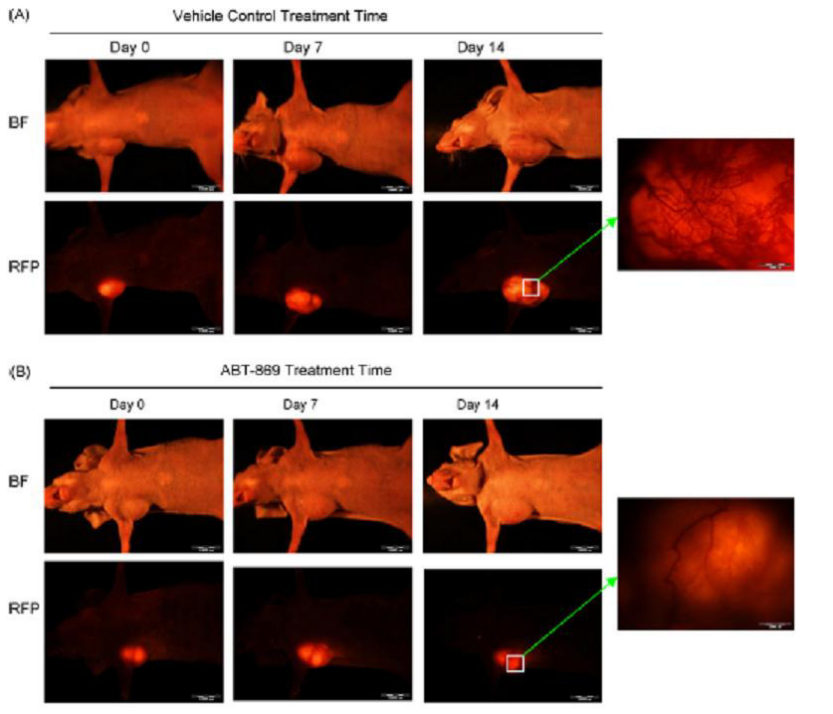
**Sequential real-time whole-body fluorescence imaging of HL60-RFP tumor growth in living mice**. (A) Mice were treated with vehicle control. (B) Mice treated with ABT-869 (15 mg/kg/day). Arrow-pointed pictures show the direct view of distribution of blood vessel network on the tumor surface in the two representative mice. There is less of a tumor vessel network in ABT-869 treated mice. BF: bright field channel. RFP: RFP channel (The picture is modified from Leukemia Research 2008; 32:1091–1100 with permission) [32].

Neoangiogenesis plays an important role in the pathogenesis of AML, so targeting VEGF/VEGFR receptors appears to be an alternative approach for treating AML [13]. Based on the early promising clinical trial results in AML patients regardless of FLT3 status achieved by other multi-targeted inhibitors like SU11248 and PTK787/ZK 222584 [29-31]. ABT-869 was also tested against a wild type FLT3-AML cell line, HL60 in a xenograft model. HL60-RFP, a stable transfectant with red fluorescence protein, was examined in both the subcutaneous and systemic leukemia xenograft models using an advanced Olympus OV100 Whole-Animal Imaging System [32]. ABT-869 reduces leukemia burden and prolongs survival of NOD/SCID mice engrafted with HL60-RFP. ABT-869 is effective in delaying tumor growth about five-fold in the subcutaneous xenograft model (Figure 7) by inhibiting angiogenesis via VEGF/VEGFRs loop [32].

## Nonclinical studies of ABT-869 as a single agent and in combination with mTOR inhibitor in Hepatocellular carcinoma (HCC)

Expression of VEGF, the primary pro-angiogenic factor, has higher in HCC than in normal hepatic parenchyma cells and has been shown to positively correlate with vascularization of HCC [33,34]. HCC cells are dependent on the supply of oxygen and nutrient through this neoangiogenesis [33,34]. Consequently, inhibition of neoangiogenesis could serve as a promising approach for the intervention of HCC.

In addition, the mammalian target of rapamycin (mTOR), a cytosolic serine/threonine kinase, has emerged as an attractive anticancer target in recent years [35]. mTOR plays an essential role not only in controlling the mammalian translation machinery, but also in regulating signaling pathways that respond to growth factors and nutrition. Activation of mTOR enhances translation of mRNAs that encodes key regulation protein for cell cycle, cell proliferation and growth such as cyclin D148 and ornithine decarboxylase 49 by phosphorylation of S6K1 (p70S6 kinase) and 4E-BP1 (EIF4-binding protein 1) [36]. mTOR is also a central downstream effector of PI3K/AKT pathways.[37] The mTOR signaling pathway has been reported to be deregulated in HCC [38,39]. Rapamycin, a mTOR inhibitor, binds to the immunophilin FKBP12, and the formed complex inactivates mTOR, further suppressing p70S6 kinase and 4E-BP1, two critical downstream targets of mTOR signaling. Rapamycin inhibits proliferation of HCC cell lines, including HepG2, Hep3B, and Sk-hep-1 [40,41]. Therefore, combining ABT-869 with rapamycin would be a reasonable targeted therapy for HCC.

We demonstrated that oral administration of ABT-869 as a single agent at a dose of 10 mg/kg/day effectively inhibits the growth of Huh7 and Sk-hep-1 tumors in mouse xenograft models [24]. ABT-869 shows a dramatic inhibition of neoangiogenesis *in vivo*. This is supported by immunohistochemistry (IHC) analysis that shows ABT-869 significantly down-regulates VEGF and reduces the formation of Microvessel density (MVD). Bevacizumab, a specific anti-VEGF antibody, was also compared with ABT-869 in a Sk-hep-1 mouse xenograft. The antitumor activity of ABT-869 is significantly higher than bevacizumab in this model [24]. Further analysis reveals that phosphorylation of p44/42 MAP kinase is also substantially decreased in the ABT-869-treated tumor samples [24]. The additional targeting achieved by the multi-targeted properties of ABT-869 could explain the significant advantage of anti-angiogenic activity of ABT-869 over bevacizumab, since MAPK pathway is known to be dsyregulated in human HCC.

Combination of ABT-869 (10 mg/kg/day) with Rapamycin (2 mg/kg/day) shows significant tumor volume reduction in both Huh7 and Sk-hep-1 animal models when compared to either of the single drug treatments (p < 0.05). Up-regulation of the cell cycle inhibitor, p27, and inhibition of the MAPK pathway contribute to the synergistic antitumor effect observed in combination therapy [24].

Taken together, these results support the rationale for clinical development of combination therapy of ABT-869 and other chemotherapies such as Rapamycin in HCC.

## Dissecting the potential resistance phenomenon in ABT-869

In contrast to their potent efficacy in cellular based assays and xenograft models, in clinical trials, FLT3 inhibitors alone only achieve moderate and transient responses in the majority of AML patients [29,42-45]. Furthermore, important experience has been gained from imatinib mesylate (Gleevec) used as monotherapy for treating chronic myeloid leukemia (CML) indicating that under prolonged therapy with TKIs, patients could develop resistance or relapse [46]. Point mutations in the ATP binding site or gene amplification of BCR-ABL are the main cause of imatinib-resistance in CML patients [47]. However, point mutations in the FLT3 kinase domain are not common [48,49].

As ABT-869 was entering early phase clinical development with continuous daily dosing schedule, we investigated some of the mechanisms that could potentially be used by leukemia cells to overcome the cytotoxic effect under long-term use of ABT-869. Three resistant cell lines (designated as MV4–11-R1, -R2, -R3) were developed by over three-month co-culture of the human leukemia cell line, MV4–11 (AML, both alleles FLT3-ITD) with increasing concentrations of ABT-869 [50]. These resistant lines are much less sensitive to ABT-869-medidated cell proliferation inhibition and apoptosis, but also are cross-resistant to structurally unrelated FLT3 inhibitors (AG1296, SU5416 and FLT3 inhibitor III). No point mutation is found in the FLT3 kinase domain in all 3 resistant lines [50]. Low density array analysis reveals that a total of 61 genes are differentially expressed more than 2-fold between the 3 resistant and parental MV4–11 cells. Interestingly, MV4–11-R cells over-express FLT3 ligand (FLT3LG) and BIRC5 (Survivin), while down-regulate the suppressor of cytokine signaling (SOCS) family (SOCS-1, -2, -3) [50]. The C-terminal domain of SOCS proteins acts as an adapter targeting kinase receptor complex for ubiquitination and subsequent proteasome-mediated degradation [51]. The SOCS family also is an important negative regulator of STAT pathways [51,52]. In MV4–11-R cells, hypermethylation silencing of SOCS genes leads to reactivation of STAT pathway activities, as evidenced by increasing levels of phosphorylation of STAT1 protein (p-STAT1), p-STAT3 and p-STAT5 [50].

Membrane-bound and soluble forms of FLT3 ligand are both biologically active [53]. FLT3 ligand plays an important role in survival, proliferation, and differentiation of hematopoietic stem and progenitor cells (HSPC) [54,55]. It has been demonstrated that the autocrine FLT3LG/FLT3 loop promotes proliferation and prevents apoptosis of primary AML blasts and AML cell lines.[56,57] Stimulation of MV4–11 cells with extra FLT3 ligand either by directly adding to the culture medium or by using conditioned medium harvested from MV4–11-R cells can further increase p-STAT1, p-STAT3, p-STAT5, as well as the expression of survivin [50], which correlate with resistance to ABT-869 and other FLT3 inhibitors (AG1296, SU5416 and FLT3 inhibitor III). On the contrary, blocking FLT3 ligand with a FLT3 ligand neutralizing antibody enhances ABT-869-induced apoptosis in MV4–11-R cells [50]. Collectively, these results indicate a prominent role of FLT3 ligand in mediating the resistance to FLT3 inhibitors.

Survivin (encoded by BIRC5), the smallest member of the inhibitor of apoptosis protein (IAP) family, has been regarded as one of the classic fetal oncoproteins [58-61]. Survivin stabilizes X-linked IAP (XIAP), another member of IAP family, against proteasomal degradation to protect cells from apoptosis [62]. To demonstrate the critical role of survivin in the regulation of resistance in MV4–11-R cells, a pool of shRNA was used to specially target survivin. Silencing survivin remarkably potentiates ABT-869-induced apoptosis in MV4–11-R cells when compared to control shRNA treatment. In contrast, forced expression of survivin in MV4–11 cells leads to resistant to ABT-869 and other FLT3 inhibitors [50].

After screening for compounds which could potentially reverse the resistance phenotype in MV4–11R, Indirubin derivative (IDR) E804 was identified. As an inhibitor of the SRC-STAT3 pathway [63], IDR E804 shows potent efficacy in re-sensitizing MV4–11-R to ABT-869. IDR E804 treatment dose-dependently induces MV4–11-R cells to undergo apoptosis and inhibits the expression of p-STAT1, p-STAT3, p-STAT5 as well as completely abolishes survivin expression [50]. In the presence of a sub-toxic concentration (2 nM) of IDR E804, the IC50 value of ABT-869 in MV4–11-R decreased from 52 to 6 nM. The combination of ABT-869 and IDR E804 also achieves better anti-tumor effect than either single agent treatment in a MV4–11-R mouse xenograft model [50].

**Figure 8:**
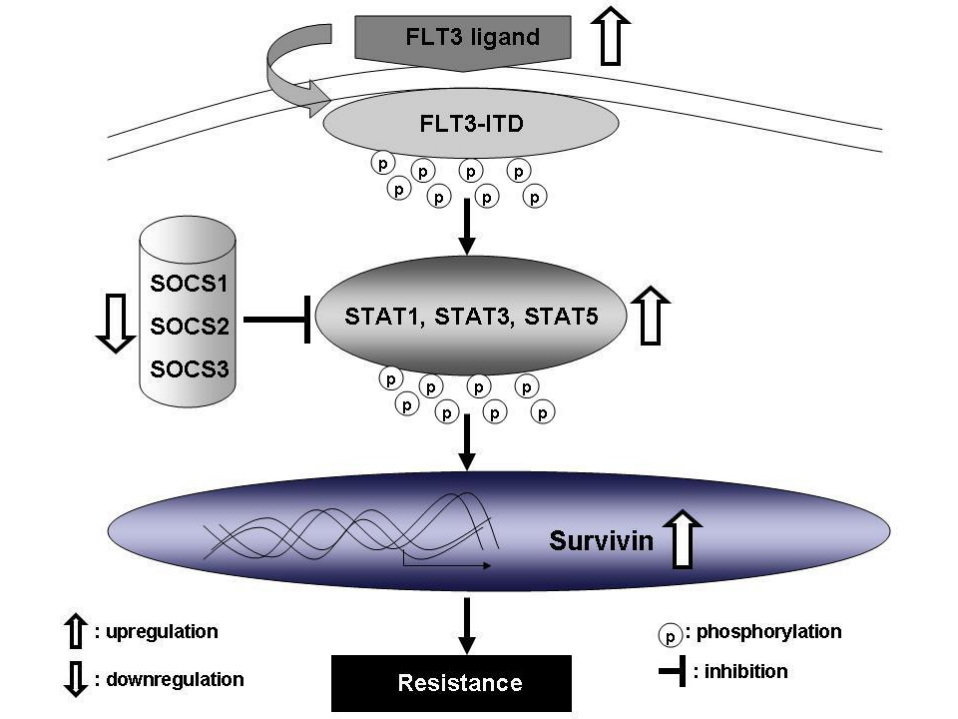
**A model of enhanced STAT activation and overexpression of survivin leading to resistant phenotype in MV4–11-R cells**. (Modified with permission from Blood journal) [50].

In summary, over expression of FLT3 ligand, methylation silencing of the SOCS family and overexpression of survivin all together integrate leading aberrant STAT signaling activity and contribute to resistance to FLT3 inhibitors. The discovery of this novel mechanism of resistance to FLT3 inhibitors, as described in Figure 8, could help develop new anti-leukemic agents or uncover compelling combinations. Combination of FLT3 inhibitors with compounds targeting the STAT pathway or survivin may represent a therapeutic strategy to minimize resistance or re-sensitize resistant cells to FLT3 inhibitors in AML patients with FLT3-ITD mutation.

## First in Man (FIM) and phase I study

In 2006, Abbott made a strategic decision and partnered with the clinical team at National University Hospital in Singapore and conducted the first in man study for ABT-869. The first in man study was started in patients with solid malignancies refractory to or for which no standard effective therapy exists who were enrolled in escalating dose cohorts and treated with oral ABT-869 once daily continuously. This study was designed as a single-arm, open-label Phase I trial and was conducted in three segments in order to determine the maximum tolerable dose (MTD), tolerability, and pharmacodynamics of a lower dose cohort to better define dose-effect relationships. ABT-869 lacks high aqueous solubility, therefore, the study drug was diluted in 60 mLs of Ensure Plus^®^. Preliminary PK at doses of 10 mg showed a modest correlation between oral clearance and body-weight; thus subsequent dose escalations in segment A were based on bodyweight. The most common drug-related adverse events were fatigue, proteinuria, hypertension, myalgia, skin toxicity (hand and foot blisters) and oral hypersensitivity, and these toxicities increased in frequency and intensity with increasing doses. The maximal tolerated dose (MTD) was determined to be 0.3 mg/kg/day. In general, the treatments are well tolerated in this patient population with either refractory disease or no standard therapy.

The treatment response of this phase I trial is encouraging. Three (10%) out of 29 patients achieved partial response (PR); two had non-small cell lung cancer (NSCLC) treated at 0.3 mg/kg/day and 10 mg/day respectively, and one had colorectal cancer (CRC) treated at 0.1 mg/kg/day. An additional sixteen patients had stable disease lasting longer than 12 weeks, among which were patients with CRC (5), NSCLC (2), ovarian cancer (2), hepatocellular carcinoma (HCC) (2) and neuroendocrine tumour (2).

**Figure 9:**
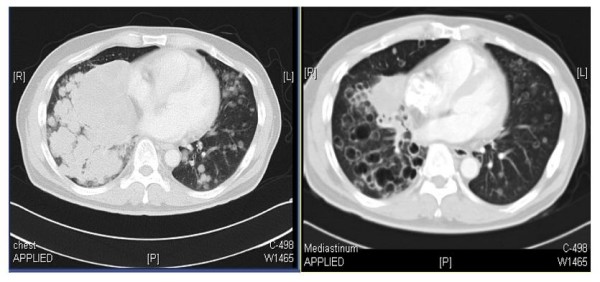
**Computed tomography scan of tumor response and cavitation of lesions in a patient with metastatic lung carcinoma showing cavitation after 2 treatment periods**. (with permission from Journal of Clinical Oncology) [64].

Tumor cavitation in the lungs and reduction of contrast enhancement in tumor on post-treatment CT scans after ABT-869 treatment suggesting central necrosis supported antiangiogenic activity, and has been observed with other VEGF antagonists (Figure 9). Prolonged stable disease lasting more than 12 months with minimal toxicity was observed in four patients; alveolar soft part sarcoma (27 months), CRC (19 months), HCC (17 months), and renal cell carcinoma (18 months) [64]. The response to ABT-869 observed in multiple tumor types suggests that histological different types of cancer could share the same dysregulated signaling pathway(s) and the rationale of multi-targeted approach may be necessary for solid tumors.

Extensive pharmacodynamic analyses were performed with this phase I trial. Exposures of ABT-869 (AUC from 0–24 h) from this trial were similar between Asian and Caucasian populations (2.7 vs. 2.3 μg·h/mL, respectively) and met the exposure targets derived from nonclinical efficacy studies [18,64]. Dynamic contrast enhanced-MRI (DCE-MRI) showed dose-dependent reduced tumor vascular permeability that correlated with drug exposure. Circulating endothelial cells (CECs) were significantly reduced (9.6 ± 7.0/μL vs. 16.5 ± 13.4/μL, p = 0.007) and vascular endothelial factor was increased (126.3 ± 104.4 pg/mL vs. 74.2 ± 82.2 pg/mL, p = 0.004) by day 15 of treatment (0.25 mg/kg) [64]. The biomarker evidence of antiangiogenic activity and DCE-MRI evidence of tumor antiangogenesis are consistent with proof of target inhibition and can be translated to observed promising clinical activity.

A multi-center phase I study was also initiated in patients with refractory or relapsed AML or myelodysplastic syndrome (MDS) as FLT-3 is an obvious therapeutic target of ABT-869. Based on our pre-clinical study [22], the trial was designed as two stages with initial monotherapy and later in combination with Ara-C. Specifically, based on our pre-clinical combination sequence data, ABT-869 will be given after the completion of Ara-C at each cycle.

## Current ongoing clinical trials

The promising anti-cancer properties of ABT-869 identified at the early phase trial facilitate further clinical development of this novel agent. In June 2007, Abbott and Genentech Inc. formed collaboration for the global research, development and commercialization for ABT-869. Phase II clinical trials evaluating ABT-869 for advanced or metastatic hepatocellular carcinoma, metastatic breast cancer, metastatic colorectal cancer, metastatic non-small cell lung cancer, and advanced renal cell carcinoma are ongoing. A summary of current ABT-869 clinical trials listed on the National Institutes of Health Website is shown in Table 2.

**Table 2:** Current listed clinical trials on ABT-869

Trial title	Enrollment	Trial design	Last verified	Recruitment	Start date
Phase 2 Study of ABT-869 in Combination With Paclitaxel Versus Paclitaxel Alone as First Line Treatment For Metastatic Breast Cancer	102	RDBT, MC	April 2009	Recruiting	March 2008
Phase 2 Study of ABT-869 in Advanced Hepatocellular Carcinoma (HCC)	44	RDBT, MC	March 2009	Active, not recruiting	August 2007
Study of ABT-869 in Combination With Tarceva in Subjects With Solid Tumors	0	W	January 2009	Withdrawn	September 2008
Phase 1 Study of ABT-869 in Subjects With Solid Tumors	24	Conducted in Japan	March 2009	Recruiting	September 2008
Phase 2 Study of ABT-869 in Subjects With Advanced Non-Small Cell Lung Cancer (NSCLC)	139	RUO, MC	March 2009	Active, not recruiting	August 2007
Phase 2 Study of ABT-869 in Combination With mFOLFOX6 Versus Bevacizumab in Combination With mFOLFOX6 as Second Line Treatment for Advanced Colorectal Cancer	102	RUO, MC	April 2009	Recruiting	August 2008
Phase 2 Study of Carboplatin/Paclitaxel in Combination With ABT-869 in Subjects With Advanced or Metastatic Non-Small Cell Lung Cancer (NSCLC)	80	RDBT, MC	April 2009	Recruiting	June 2008
Phase 2 Study of ABT-869 in Subjects With Advanced Renal Cell Carcinoma Who Have Previously Received Treatment With Sunitinib	53	Open label, NR	April 2009	Active, not recruiting	August 2007
Phase 2 Study of Oxaliplatin, Fluorouracil, Leucovorin and ABT-869 or Bevacizumab as Second-Line Therapy in Treating Patients With Locally Recurrent or Metastatic Colorectal Cancer	0	Single center	October 2008	Not yet recruiting	October 2008
Phase 1 Pharmacokinetic Study To Evaluate Effect of Food and Diurnal Variation on ABT-869	12	Single center	March 2009	Recruiting	February 2009

Data compiled from

RDBT: Randomized, placebo-controlled, double blind trial

MC: Multicenter

W: Withdrawn prior to recruitment

RUO: Randomized, uncontrolled, open label

NR: Non-Randomized

Preliminary clinical data on single agent ABT-869 was presented in the 2009 ASCO annual meeting. Encouraging clinical activity has been observed in non-small cell lung cancer (NSCLC) and advanced hepatocellular carcinoma (HCC) trials as well as in a renal cell carcinoma (RCC) trial after Sunitinib failure [65-67]. However, additional studies are required to determine the optimal dosing strategy especially in RCC and HCC patient population as frequent dose interruption or reduction was observed. In the NSCLC trial, two different doses were tested (0.10 mg/kg and 0.25 mg/kg), and preliminary data did not show significant difference in OS and PFS between these two arms. Furthermore, current pharmacokinetic analysis indicates that body weight does not significantly impact exposure suggesting that a fixed dosing strategy may be appropriate [68].

## Conclusions and future directions

In summary, ABT-869 is a novel inhibitor that simultaneously provides potent and selective inhibition of the VEGFR and PDGFR kinase families and has demonstrated activity in patients with solid tumors who failed standard regimen. Optimal dosing and scheduling are being investigated and the potent *in vivo *angiogenesis effect has already produced a promising clinical response in early phase clinical development.

Based on the Population PK analysis presented in an abstract [68], ABT-869 PK fits one-compartment model with first order absorption and elimination. Race, sex and impaired renal function do not appear to significantly affect PK. In addition, body weight does not significantly impact exposure suggesting that a fixed dosing strategy may be appropriate.

The reported side effects such as fatigue, proteinuria, hypertension, myalgia, skin toxicity (hand and foot blisters) are similar to commonly described toxicity in other FDA approved oral tyrosine kinase inhibitors such as Sunitinib. Long term dosing of ABT-869 did not appear to pose problems of cumulative toxicity in patients who received more than a year of dosing. The nonclinical studies on combination therapies have demonstrated synergy and are likely to be more effective than monotherapy. Clinical studies of ABT-869 in combination with chemotherapy or other novel targeted therapies, will further our understanding of how to optimize this exciting new therapy. The recent identification of the critical role of survivin in the regulation of ABT-869 resistance is interesting and is therapeutically relevant. Mechanisms of resistance to ABT-869 remain under active investigation.

## Competing interests

DHA is an employee of Abbott laboratories. The other authors declared no conflict of interest.

## Authors' contributions

JZ wrote the review of pre-clinical studies. BCG contributed the phase I trial data, and DHA contributed non-clinical studies, discussion and revision. CSC conceived of the study, wrote the review of clinical trials and organized the manuscript. All authors read and approved the final manuscript.
